# Pyrosequencing-Based Transcriptome Analysis of the Asian Rice Gall Midge Reveals Differential Response during Compatible and Incompatible Interaction

**DOI:** 10.3390/ijms131013079

**Published:** 2012-10-12

**Authors:** Deepak Kumar Sinha, Javaregowda Nagaraju, Archana Tomar, Jagadish S. Bentur, Suresh Nair

**Affiliations:** 1 Plant Molecular Biology Group, International Centre for Genetic Engineering and Biotechnology, Aruna Asaf Ali Marg, New Delhi 110067, India; E-Mail: deepak22sinha@yahoo.co.in; 2 Laboratory of Molecular Genetics, Centre for DNA Fingerprinting and Diagnostics, Hyderabad 500001, India; E-Mail: archanat@cdfd.org.in; 3 Directorate of Rice Research, Rajendranagar, Hyderabad 500030, India

**Keywords:** *Orseolia oryzae*, susceptible host, resistant host, next generation sequencing (NGS), real time PCR, insect biotypes, insect-plant interaction

## Abstract

The Asian rice gall midge (*Orseolia oryzae*) is a major pest responsible for immense loss in rice productivity. Currently, very little knowledge exists with regard to this insect at the molecular level. The present study was initiated with the aim of developing molecular resources as well as identifying alterations at the transcriptome level in the gall midge maggots that are in a compatible (SH) or in an incompatible interaction (RH) with their rice host. Roche 454 pyrosequencing strategy was used to develop both transcriptomics and genomics resources that led to the identification of 79,028 and 85,395 EST sequences from gall midge biotype 4 (GMB4) maggots feeding on a susceptible and resistant rice variety, TN1 (SH) and Suraksha (RH), respectively. Comparative transcriptome analysis of the maggots in SH and RH revealed over-representation of transcripts from proteolysis and protein phosphorylation in maggots from RH. In contrast, over-representation of transcripts for translation, regulation of transcription and transcripts involved in electron transport chain were observed in maggots from SH. This investigation, besides unveiling various mechanisms underlying insect-plant interactions, will also lead to a better understanding of strategies adopted by insects in general, and the Asian rice gall midge in particular, to overcome host defense.

## 1. Introduction

Insects are exemplars in adapting to the ever-changing environment and have demonstrated explosive evolutionary success in terms of number of species. Their adaptability and phenotypic plasticity is the result of enormous genetic and phenotypic diversification allowing them to overcome wide range of challenges during their short life span [1]. Among the highly evolving insect families, Cecidomyiidae, termed as gall midges, is a family of flies, most of which feed within the plant tissue creating abnormal plant growths called galls [2].

Among the plant-feeding Cecidomyiids, the Hessian fly (*Mayetiola destructor*), Orange wheat blossom midge (*Sitodiplosis mosellana*), Sorghum midge (*Stenodiplosis sorghicola*), the Asian rice gall midge (*Orseolia oryzae*) and the African rice gall midge (*Orseolia oryzivora*) are agriculturally important gall midges reported to cause huge economic loss to field crops [3]. The Asian rice gall midge and the African rice gall midge, members of the *Orseolia* genus (comprising of 21 species), are serious insect pests of rainfed and irrigated lowland rice causing high yield loss in rice growing areas [4].

Adult flies of rice gall midge are mosquito-like and live for only few days. Females mate only once and lay about 100 to 150 eggs on the leaf-sheath of the rice plant. These eggs hatch on the fourth day and the newly hatched maggots crawl down the space between the leaf sheaths to reach apical meristem for feeding. High humidity and presence of thin film of water on the plant surface facilitate maggot movement to the meristem. Maggots lacerate the meristem tissue with pharyngeal spatula and feed on the oozing cell sap. Laceration and secretion of saliva results in hypertrophy and hyperplasia of cells, that leads to development of a gall chamber surrounding the maggot [5,6]. The midge feeds actively for two weeks, and molts twice before pupating. With cessation of maggot feeding, the gall chamber rapidly elongates and protrudes out. The pupa wriggles up to the tip of the gall, drills an exit hole and partly pushes out of the gall. The adult emerges through eclosion of the puparium [3,4].

The gall midge-rice interaction is characterized by either a compatible or an incompatible interaction. In a compatible interaction, the virulent maggots feed on the susceptible host (SH) varieties that lead to the formation of silver shoot or leaf sheath gall in the host resulting in sterility of the tiller. During an incompatible interaction the maggots trying to feed on resistant host (RH) varieties fail to establish and finally succumb. Varietal differences in resistant host account for the differences in the defense response [hypersensitive (HR+) and non-hypersensitive (HR-) mediated defense response] against the gall midge. HR+ type defense response in the host is characterized by tissue necrosis at the site of feeding while the HR- type defense response in the host is not manifested with a necrotic site. However, both the defense responses lead to mortality of the maggots [7].

The general strategy to manage rice gall midge is through breeding resistant crop varieties. Plant resistance to gall midge is a genetic trait and various sources of resistance are available in the rice germ plasm. There are over 60 gall midge-resistant varieties of rice that have been bred and released for cultivation [4]. However, cultivation of resistant rice varieties to manage the pest is prone to certain limitations. One such limitation is the breakdown of resistance, hence lack of durability. Breakdown of resistance occurs as gall midge resistance in rice is governed by a single dominant gene and due to the widespread cultivation of these resistant varieties with single resistance gene. The gall midge problem is compounded by the fact that there are many biotypes of this insect and new biotypes are continuously evolving [6,8]—A major reason for breakdown of host resistance genes. Therefore, in order to devise effective strategies for management of gall midge, a better understanding of the molecular mechanism of defense in the rice host and also the corresponding survival strategies adopted by the insect, is necessary. Hence, there is an urgent need to understand the molecular basis of gall midge-rice interaction considering both the facets that include plant defense and antagonistic co-evolution of insect virulence.

Basic information on the gall midge-rice interaction is limited and also there is lack of substantial molecular resources to study this destructive gall-forming pest. Therefore, results emanating from functional genomics study will greatly help fill this gap and serve as a valuable resource for designing future studies to gain important insights into this interaction. Recently, few genes from the rice gall midge have been cloned and have been shown to be involved in gall midge-rice interaction [9,10]. In this regard, we employed the Roche 454 next generation sequencing (NGS) technique to develop transcriptomic and genomic resources for this pest. The NGS technologies offer a prime opportunity in generating molecular resources that will prove useful in understanding gall midge-rice interaction in particular and insect-plant interaction in general.

Roche-454 NGS technology has revolutionized the arena of genomics for non-model species that have little or no previously existing sequence information. The 454 pyrosequencing technique produces longer fragments and is thus well suited for less studied species. The 454 pyrosequencing strategy has been employed to unveil molecular signatures in varied insects such as *Manduca sexta* (hornworm) [11], *Bemisia tabaci* (white fly) [12], *Aphis glycines* (soybean aphid) [13] and *Agrilus planipennis* (emerald ash borer) [14]. However, this is the first study that focuses on whole transcriptome changes in the Asian rice gall midge (*Orseolia oryzae*) biotype 4 (GMB4) feeding on susceptible or resistant (HR+ type) rice varieties. Besides, this is also the first report of a comparative study of transcriptomes from a single biotype of an insect involved in two different interactions with two different genotypes (resistant and susceptible) of its host. This study would also generate molecular resources for the identification of candidate genes involved in gall midge-rice interaction. Additionally, the data generated will help in identification of EST-based molecular markers and single nucleotide polymorphism (SNPs) that can be utilized for gall midge biotype differentiation. Furthermore, with the development of diagnostic markers identification of endosymbionts present/integrated in the gall midge genome can be performed.

## 2. Results and Discussion

### 2.1. Pyrosequencing and Assembly

**Figure 1:**
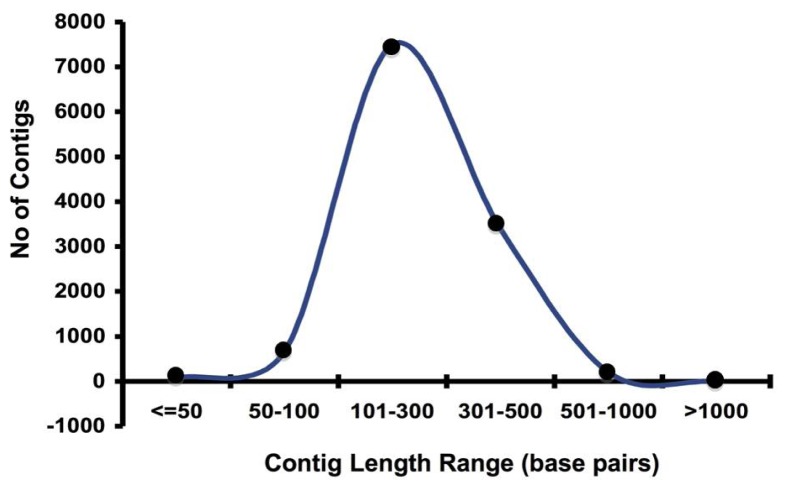
Contig length range of the assembled contigs represented in the Asian rice gall midge (GMB4) library.

**Figure 2:**
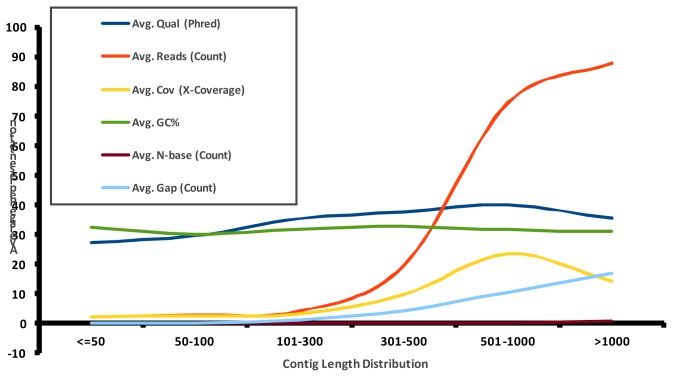
Contig length distribution of the Asian rice gall midge (GMB4) transcripts from 454 sequencing. The red, green, blue and brown lines denote number of average reads, average percentage GC content, average gap count and average N-base count, respectively.

454-Pyrosequencing has made the field of genome wide transcriptome studies conceivable in non-model organisms with very little prior genome sequence information [15]. Our current study has contributed a large number of expressed sequences tags (ESTs) from the Asian rice gall midge through 454-GS-FLX pyrosequencing. This investigation, after removal of adaptors and low complexity sequences, resulted in 79,028 EST sequences from GMB4 feeding on susceptible host (SH) rice variety TN1 and 85,395 sequences from GMB4 feeding on resistant host (RH) rice variety Suraksha. The detailed description of general features of EST libraries prepared from GMB4 from SH and RH is provided in Table 1. Additionally, in order to increase the transcriptome coverage and to facilitate correct assembly, the filtered sequences of both the libraries were assembled and used as a backbone for further analysis. The total numbers of available reads from both the samples were, 161,081 out of which 71.2% could be assembled into contigs. The total numbers of contigs generated were 11,858 with average contig length of 263 bp. Post filtering, the assembly statistics, contig length range and contig length distribution are shown in Table S1, Figures 1 and 2, respectively.

### 2.2. BLAST and Gene Ontology Analysis

**Figure 3:**
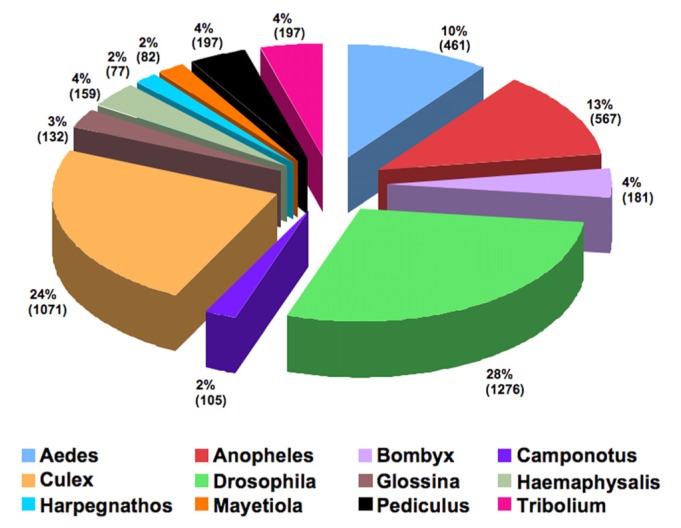
A pie chart distribution depicting top BLASTX hits of the Asian rice gall midge transcripts to various arthropods.

The sequenced ESTs were subjected to BLASTX homology search against the NCBI nr database. In the combined assembly, only 18,817 transcripts out of total 58,247 (32.3%; 11,858 contigs and 46,389 singlets) transcripts showed homology with sequences present in the NCBI database. Out of these only 5006 transcripts showed significant homology with E-value cutoff of ≤ 10^−3^. Alternatively, all these sequences were also annotated with the UniRef90 database [16]. The UniRef database enables the complete coverage of sequence at high resolution and conceals redundant sequences. This database includes a clustered set of sequences from the UniProt Knowledgebase (UniProtKB) and UniProt Archive records. Decrease in redundancy reduces sampling bias and over-representation of sequences, thereby increasing the speed of homology-based searches and correct detection of distant relationships [17]. Among the EST sequences (11,858 contigs and 46,389 singlets) of both the samples (maggots from SH and RH, respectively), 5,570 (9.56%) showed significant homology (E-value cutoff ≤ 10^−3^) with the annotated sequences in the UniRef database. BLASTX results obtained using UniRef90 or NCBI nr as reference databases yielded hits that were 95% common. The remaining sequences (52,677; 90.44%) exhibited insignificant matches (E-value cutoff > 10^−3^). It may be noted that a huge subset of sequences did not share similarity with sequences in the public database. This suggests that a large majority of the rice gall midge EST sequences are yet to be assigned a putative function and this may be a direct consequence of dearth of diverse insect whole genome annotation in the database. Top 12 species exhibiting maximum similarity with the gall midge sequences are shown in the Figure 3. The gall midge belongs to the order Diptera and therefore predictably a high degree of similarity was observed with the sequences from other dipteran insects (*Drosophila*, 28%; *Culex*, 23.7%; *Anopheles*, 12.5%; and *Aedes*, 10%).

**Figure 4:**
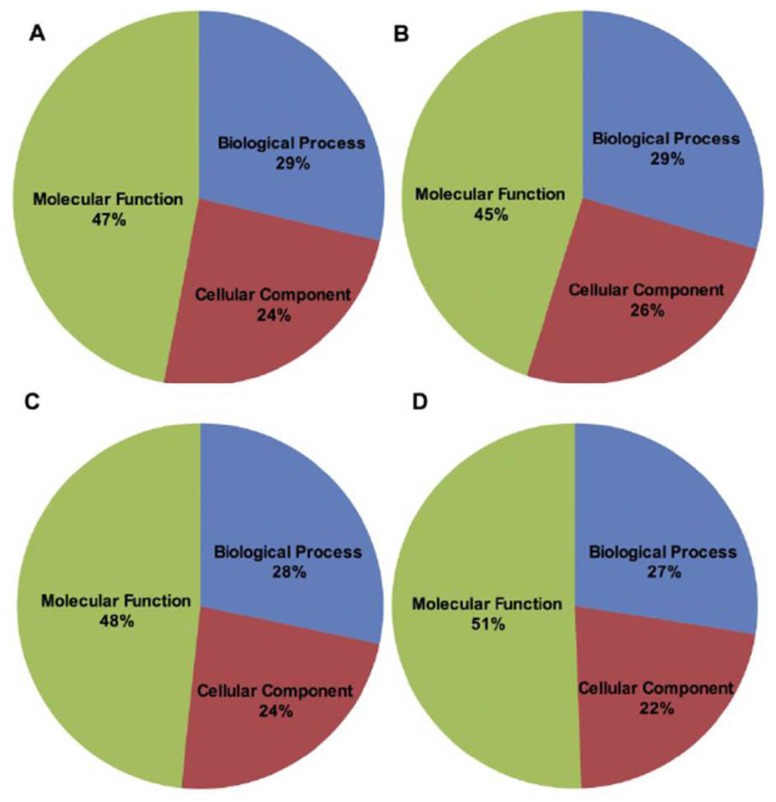
Gene ontology distribution of the transcript sequences of Asian rice gall midge. Percentage of transcripts categorized into biological process, molecular function and cellular components of (**A**) all sequences (includes pooled transcripts of both GMB4 maggots from SH and RH) (**B**) transcripts commonly present in both GMB4 maggots from SH and RH **(C**) specifically present in GMB4 maggots from SH and, (**D**) specifically present in GMB4 maggots from RH.

All (maggots from SH and RH) sequences showing matches to known sequences in the public domain databases were assigned Gene Ontology (GO) categories that included biological process, molecular function and cellular components (Figure 4A). Similar categories were also assigned to sequences (i) commonly present in both maggots from SH and RH (Figure 4B); (ii) specifically present in maggots from SH (Figure 4C) and, (iii) specifically present in maggots from RH (Figure 4D). Results presented in Figure 4A revealed the total genetic makeup of the biotype of gall midge used in the present study; in Figure 4B sequences commonly present in both maggots from SH and RH are shown. These are likely to identify ESTs coding for genes, amongst other functions, responsible for virulence. In contrast, results depicted in Figure 4C,D revealed genes that are specifically expressed by the midge depending on the host type (SH or RH) it encounters. Furthermore, these results revealed alterations in the transcriptome of the insect brought about by the nature (susceptible or resistant) of the host.

### 2.3. Metabolic Pathway Analysis

The sequences from SH and RH samples were analyzed for their representation in various metabolic pathways using KEGG server [18]. Overall, 1025 transcripts from SH and 935 transcripts from RH were assigned to 172 KEGG metabolic pathways (Table S2). The maximum number of transcripts was found in Ribosome (SH: 76 and RH: 77) followed by oxidative phosphorylation metabolic pathway (SH: 58 and RH: 51). Metabolic pathways such as Glycosphingolipid biosynthesis-ganlio series, Diterpenoid biosynthesis, ABC transporter, Bacterial secretion system and Notch signaling pathway were not represented in the SH sample. Metabolic pathways such as valine, leucine and isoleucine biosynthesis, steroid biosynthesis, ubiquinone and other terpenoid-quinone biosynthesis and Jak-STAT signaling pathways were not represented in RH sample. Our results also revealed differences in the amino acid biosynthesis pathway between the two samples. We suggest that these observed differences including aminoacyl-tRNA biosynthesis (Figure S1A,B) pathway in both SH and RH samples could be as a result of maggots feeding on two different types of host have different amino acid requirements for their survival. This hypothesis regarding the differences observed in amino acid and aminoacyl-tRNA biosynthesis (Figures S1A,B) pathways in both SH and RH samples is further borne out by the fact that previous studies [19] involving the Hessian fly-wheat interaction reported manipulation of amino acid content of the host by virulent Hessian fly larvae. Differences in the oxidative phosphorylation metabolic pathway were also observed between the SH and RH samples (Figure S2A,B). Summing up, this analysis has revealed differential response of the maggots feeding on two different hosts (one susceptible and the other resistant). Even though not all major genes from different pathways were identified by the KEGG analysis, the data generated will, nonetheless, provide a good starting-point for initiating studies on pathways important in the gall midge-rice interaction.

### 2.4. Genes of Interest

The present investigation has generated a large database of genes from the gall midge. Some of the identified genes have been categorized and listed in Table 2. Our data included transcripts from metabolic activities such as protein phosphorylation, induction of immune regulatory proteins, representation of wide range of proteases, prevalence of cell death related genes and genes coding for antioxidants. The alterations at genic level, with detailed function and probable involvement of the identified transcripts in the interaction are sub-grouped and discussed below.

#### 2.4.1. Immune Regulatory Proteins

Extensive mining of the data sets led to the identification of putative genes reported to be involved in signal transduction, immunity and melanization. The Asian rice gall midge, during a compatible interaction, spends a major part of its life cycle inside the host. Therefore, immune regulatory proteins are probably required by the maggots to overcome the constant challenge posed by host plant’s defense machinery. In insects, innate immune response against pathogens involves membrane bound molecules such as pathogen recognition receptors (PRRs), induction of proteolytic cascade (induction of Toll signaling pathway) resulting in the synthesis of anti-microbial peptides, haemolymph clotting and melanization at the localized sites [20]. Galectins, as PRR, have been widely implicated in the identification of membrane bound molecules both in the insect and mammalian system [20,21]. Our investigation has identified galectin (S-type lectin) and mannose-binding lectins in the library. Also, genes related to proteolytic cascade and signal transduction such as Toll, ubiquitin carrier protein, histone H2A and transcription factor NFAt were identified (Table 2). However, it is still not clear if residence inside the host-plant leads to the induction of genes of the proteolytic cascade in the maggots. Antimicrobial peptide such as lysozyme c-1 was also represented. Besides, the mode of action of these peptides is not yet fully understood. It is pertinent to mention that insect immunity can at best be described as a general response. And therefore, it is quite likely that it may be even triggered by the presence of an abiotic stress factor generated by the host. Melanization, as stated above, is the final arsenal in the insect defense system [22] and in this investigation, genes associated with this process such as prophenoloxidase, dihydropteridine reductase, serpins and laccase have also been identified (Table 2). It is plausible that these genes have a role to play in the insect during incompatible interaction, because reddish dark pigmentation (melanization) occurs in maggots feeding on the resistant plant in which the gall midge maggot finally succumbs.

#### 2.4.2. Proteases

In order to win the “arms race” against the host plant, pests devise strategies that include “resistance by avoidance of plant toxins” and “metabolic resistance to toxins” [1]. Although evolutionary factors resulting in such an adaptation in pests against plant toxins are not fully understood, the induction and involvement of different classes of proteases has been predicted (and proved in some cases) that enables the pests to optimize their fitness with respect to the alterations in the toxin level [23–26]. Several effector virulence genes reported in pest-plant interactions include proteases such as cysteine proteases (*Avr* genes from *Xanthomonas campestris*) [27] and metalloproteases (*Avr* genes from *Magnaporthae grisae*) [28]. Also, serine, cysteine, aspartic and threonine proteases are the three defined endopeptidases with known roles in insect immunity, digestion and molting [29]. Cysteine proteases such as cathepsin, play an important role in defense system by eliminating the foreign proteins [30], protein degradation during food digestion [31], embryogenesis [32] and metamorphosis [33]. Cathepsin L, serine protease H2, CLIP domain containing protease, aspartic proteases, cathepsin D aspartic proteases were also represented in our EST library (Table 2). Serine proteases such as trypsin and chymotrypsin have been identified and cloned from the wheat midge, Hessian fly [34] and also by us in our earlier studies [10] suggesting their probable role in insect-plant interaction.

#### 2.4.3. Protein Kinases

Protein kinases account for approximately 2% of the genes in a eukaryotic genome [35]. They modify the activity, location and affinity of the substrate protein by phosphorylation and alter cellular, signal transduction and coordination processes [36]. These alterations result in differences in the signal transduction, immunity and stress response. Kinases such as MAP kinase, serine-threonine kinase have also been reported to be acting as regulators of virulence in fungal and bacterial pathogens [37]. The list of protein kinase transcripts identified included, serine-threonine kinase, MAP kinases, inositol polyphosphate multikinase, nucleoside diphosphate kinase, casein kinase and tyrosine kinase (Table 2). Serine-threonine kinases have been reported to be involved in toll signaling pathway thereby in insect immunity [38]. MAP kinase, serine-threonine kinases and phosphatase have been proved to be acting as virulence factors in different interactions [37]. MAP kinase signaling pathway has also been reported to confer resistance against endoplasmic reticulum stress [39]. MAP kinases and tyrosine kinases have been reported to play crucial role in toll signaling cascade and ecdysteroid biosynthesis [40]. These have also been reported to be involved in conferring freeze tolerance in insects [41]. The protein kinases identified in this study suggested a complex cascade of protein phosphorylation and dephosphorylation events occurring within the insect when encountered with two different sets of environment—one inside a susceptible host and the other inside a resistant host. However, to decipher the precise role of these kinases, further studies are needed.

#### 2.4.4. Genes Involved in Apoptosis

Apoptosis or programmed cell death is exquisitely regulated and is an obligatory event in multicellular organisms required for the elimination of potentially harmful or unnecessary cells either in normal development or homeostasis (both internal and external environment of the cell). Genes related to apoptosis have been reported to be involved in Hessian fly-wheat interactions [42]. The current study identified several genes predicted or shown to be involved in programmed cell death in insects. Some of these genes included caspase, inhibitor of apoptosis 2, defender against apoptotic cell death (DAD1) and programmed cell death protein 4a (Table 2). The presence of genes encoding apoptotic proteins can be explained by the fact that maggots feeding on a resistant host are probably under stress, due to their inability to sustain feeding, due to resistance gene product(s), challenge from reactive oxygen species released by the host on encountering the maggot and, exposure to different host allelochemicals and thereby initiating a series of events that eventually leads to apoptosis or programmed cell death.

#### 2.4.5. Genes Related to Reactive Oxygen Species (ROS) Pathways

Phytophagous insects are consistently challenged by reactive oxygen species and have to cope with the ROS derived from exogenous (from host) and endogenous sources [43]. When a plant is exposed to an invading insect, it mounts a defense response that includes production of ROS. The phytophagous insect tries to elude this defense mechanism mounted by the host, and produces antioxidant molecules to counter the host defense. Also, a complex cascade of antioxidant defense is initiated within the insect due to the endogenous stress and also the exogenously present pro-oxidant allelochemicals or xenobiotics produced by the host in response to herbivory [43]. Therefore, a cluster of antioxidant proteins such as catalases, cytochrome P450, superoxide dismutase and glutathione-*S*-transferase are produced to detoxify these molecules. The current investigation has also identified several anti-oxidizing enzymes such as glutathione peroxidase, Cu-Zn superoxide dismutase and catalase (Table 2). The prevalence of these transcripts in both the maggots feeding on SH and RH suggests that there is constant production of ROS by the plant upon gall midge infestation and consequently antioxidants are produced inside the gall midge in both the interactions to overcome the stress imposed by the host. The gall midge successfully avoids these molecules in a compatible interaction and is probably unable to overcome the oxidant defense response in an incompatible interaction and finally dies.

### 2.5. Detection of Molecular Markers

There is an immediate need for molecular markers that can be used for biotype differentiation and also for tagging and mapping of virulence genes in the rice gall midge. Pyrosequencing generated a large set of markers that includes EST-simple sequence repeats (EST-SSRs) and single nucleotide polymorphism (SNPs). All the contigs and the singlets identified in the present study were screened for the presence of di-, tri-, tetra-, penta-, hexa- and complex nucleotide repeats. We identified 4218 microsatellite repeats and the summary of the results is provided in Table 3. Majority of microsatellite loci comprised of dinucleotide repeats (56.4%) followed by trinucleotide repeats (28.5%). The loci thus identified were also analyzed for primer designing and primers were designed for 2,303 loci (data not shown). For identification of SNPs, we analyzed contigs and identified 2756 putative SNPs in various contigs. Tables 4 and S3 provide details of the SNP types identified. Full details of designing and validation of the primers for SSRs and SNPs are in progress and will be published elsewhere.

### 2.6. Comparative Analyses of the Asian Rice Gall Midge Transcripts with the Hessian Fly

Comparative genomics of the gall midges will help in deciphering the evolutionary relationships between them and also guide us in selection of genes or proteins that can be targeted for development of a comprehensive IPM strategy. The Hessian fly is the most studied species among the gall midges and phylogenetically one of the nearest neighbors of the Asian rice gall midge. BLASTn analysis of the ESTs from the Asian rice gall midge resulted in 9125 ESTs (E-value cutoff ≤ 10^−3^) showing similarity with the sequences from the Hessian fly (Table S4). Out of these 57.3% and 42.6% sequences were homologous to sequences from the Hessian fly midgut and salivary glands, respectively. These data suggested conservation of several transcripts present in both midgut and salivary glands of the gall midges irrespective of the differences in the hosts on which they feed. There is substantial evidence to suggest that secretions from the salivary gland and midgut in plant feeders contain proteins with regulatory roles and detoxifying ability that also act in suppressing host’s defense and altering host physiology [44]. Therefore, these identified transcripts are of great importance with respect to plant-insect interaction studies and can be targeted for manipulation with the aim of managing the pest.

### 2.7. Comparative Analyses of Transcripts

There are very few comparative studies that reveal global transcriptome changes in an insect feeding on two different genotypes of the host [45]. This study compares molecular changes that occur in one biotype of the Asian rice gall midge feeding either on (a) susceptible (TN1; compatible interaction) or on (b) resistant (Suraksha; incompatible interaction) rice variety. Basic understanding of host resistance mechanisms and also the way in which the pest reacts to different hosts will help elucidate different facets of the interactions.

**Figure 5:**
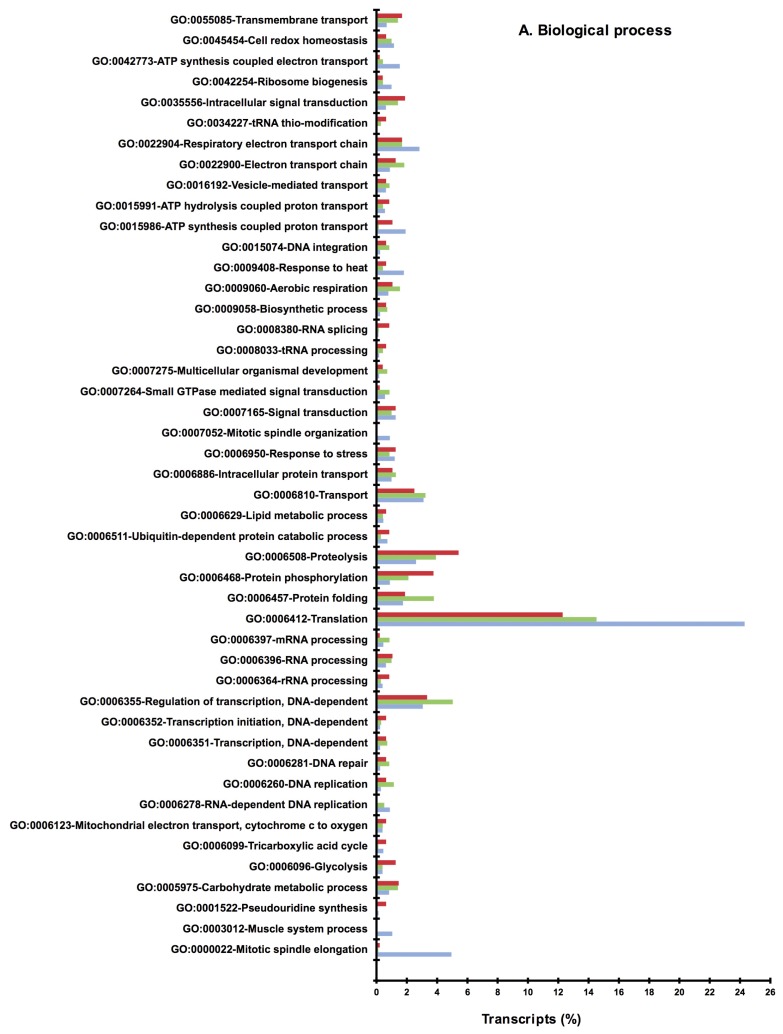

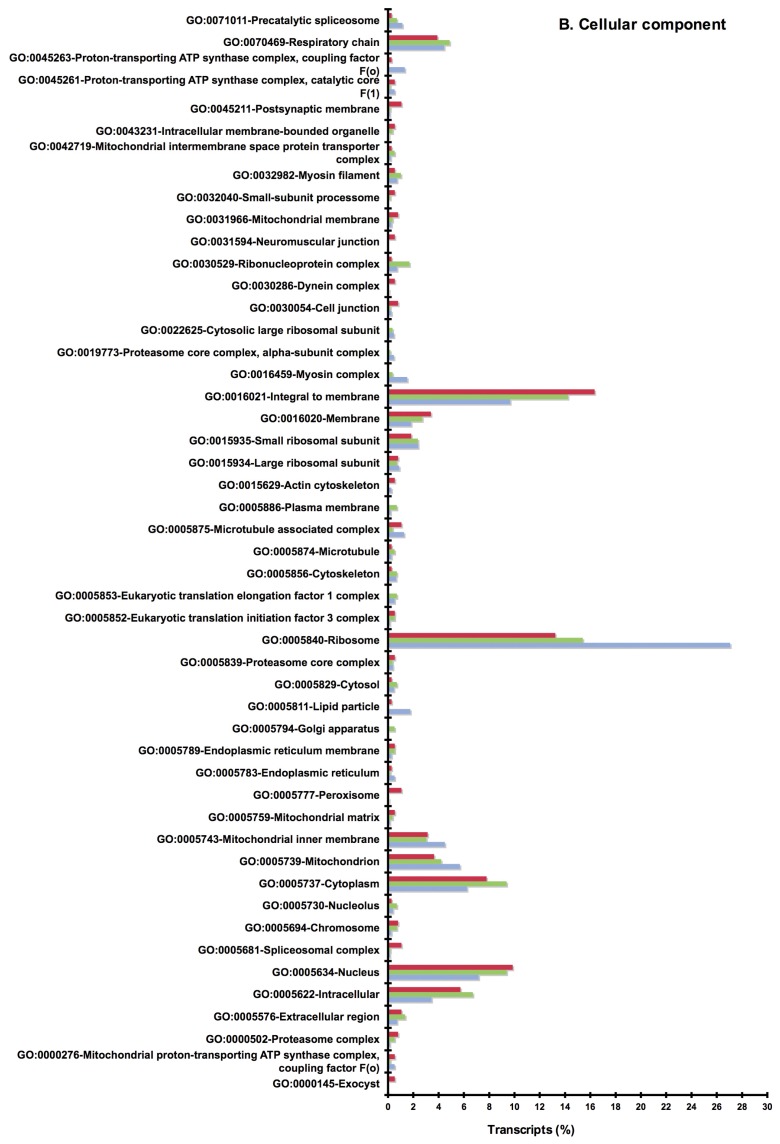

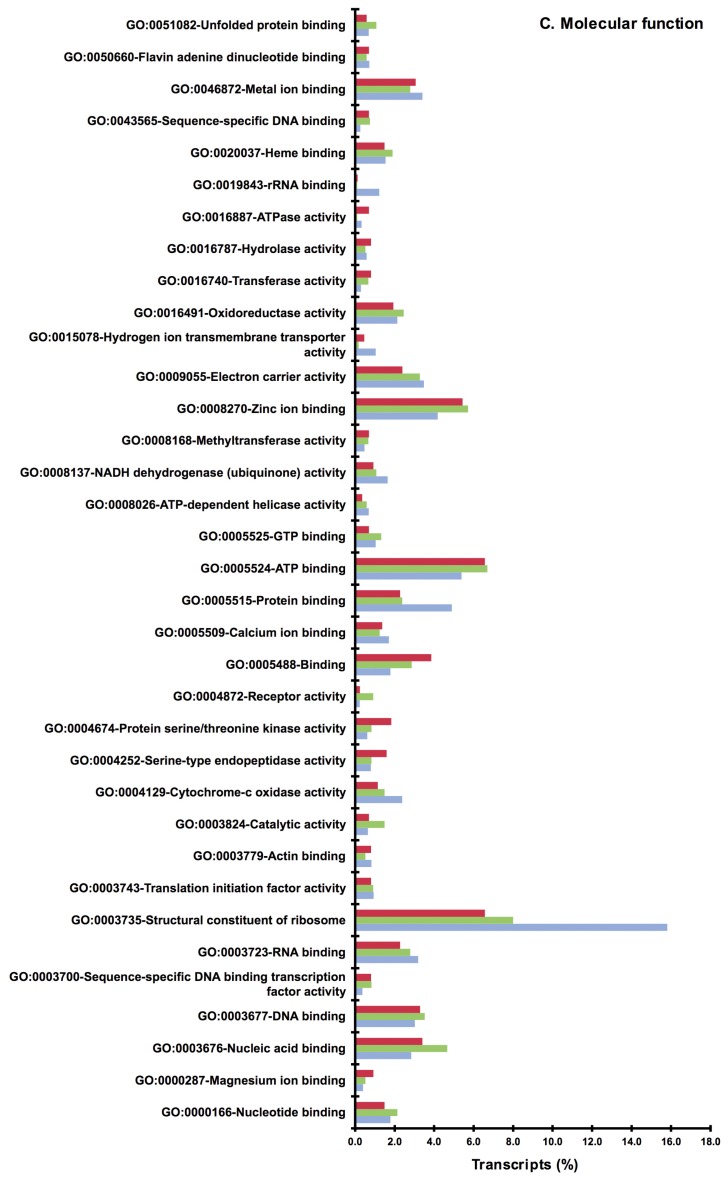
Comparison of transcript distribution of gene ontology terms. Percentage (based on the total number of transcripts in each category *i.e.*, SH, RH and Common) of transcripts specifically present in GMB4 maggots from SH (green bars), RH (brown bars) and commonly (blue bars) present in both the samples categorized in different GO terms of (**A**) biological process; (**B**) molecular function and (**C**) cellular components.

*In-silico* subtraction of annotated sequences from SH and RH samples yielded 1171 unique transcripts in maggots from SH and 952 unique transcripts in maggots from RH (Figure S3). A total of 1542 transcripts were present in both the samples. GO classification of all the transcripts was carried out separately for each group (maggots from SH, RH and common ESTs) and Figure 5 shows the distribution of GO terms in maggots from SH and RH samples and also those ESTs that are commonly present. A large number of sequences that did not show significant hits with any of the sequences in the database were considered coding for uncharacterized proteins, small or non-coding RNAs or products from endosymbionts. It is noteworthy to indicate that our earlier studies reported the presence of endosymbiont *Wolbachia* in Asian rice gall midge [46]. These sequences are currently being analyzed for endosymbionts as well as for non-coding RNAs.

All the transcripts identified from above (SH and RH) have been assigned GO terms separately and presented in Figure 5 (A: Biological properties; B: Cellular components; C: Molecular function). Transcripts involved in Pseudouridine synthesis were observed only in maggots from RH (Figure 5A). Also, transcripts of protein phosphorylation and proteolysis were relatively abundant in these maggots. Further, this set had abundance of genes involved in protein serine-threonine kinase and serine type endopeptidase activity (Figure 5C). When the maggots’ attempt to infest the host fails, it leads to events of proteolysis finally resulting in maggot mortality [10]. In contrast, comparatively higher number of transcripts involved in translation, regulation of transcription and electron transport chain were observed in maggots feeding on the susceptible rice variety (Figure 5A). Also, transcripts encoding structural constituents of ribosome, nucleic acid binding (Figure 5C, Molecular functions), ribosome, ribonucleoprotein complex and cytoplasm (Figure 5B, Cellular component) were more represented in this set. Presence of these transcripts suggests that in compatible interaction the maggots successfully orchestrate the plant metabolic machinery and subvert the plant cell’s natural functions for their own ends. However, this will need to be validated through quantitative real time PCR.

It is known that due to the unsaturated nature and short fragment size of the ESTs, an inherent shortcoming of pyrosequencing, quantitative estimation drawn from such EST data can be erroneous. Therefore, to overcome this limitation contigs that were longer than 250 bp were considered for this analysis. The present investigation revealed a set of genes that were found to be specifically present in GMB4 from SH or RH (Table S5). The 15 contigs with maximum representation in both samples and uniquely present in GMB4 maggots from either SH or RH are tabulated in Table 5. However, all these genes will have to be validated for their expression and specificity of action for elucidating their exact role in gall midge-rice interaction.

### 2.8. Expression Profiling by RT-PCR

Quantitative real time PCR was performed to study 10 selected genes of *Orseolia oryzae*. These genes were selected based on the earlier reports [37] of their involvement/modulation in pest-plant interaction. All the selected genes showed differential expression in both SH and RH samples.

**Figure 6:**
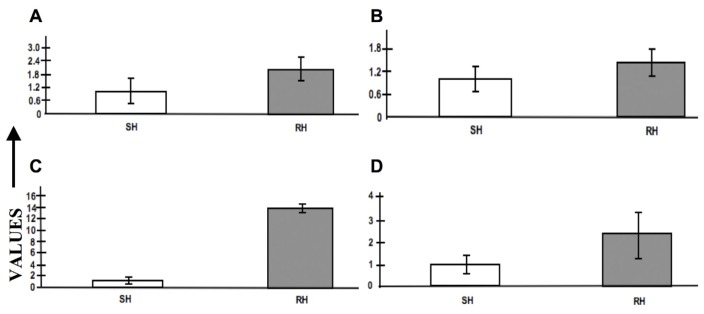

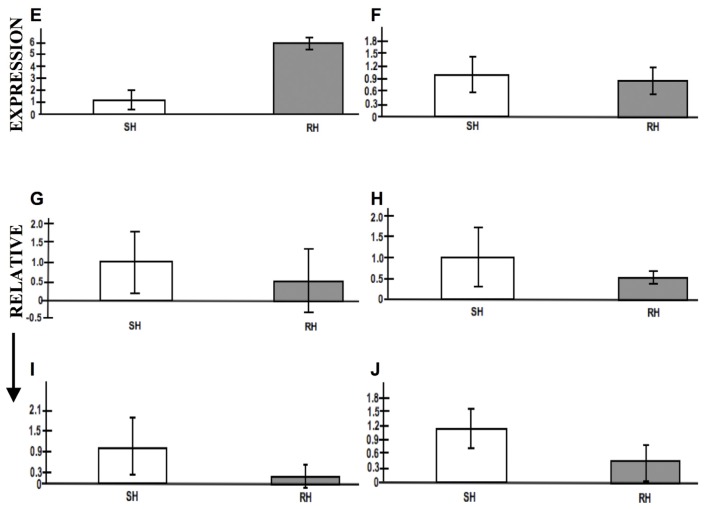
Relative expression profiles of 10 selected genes in the gall midge maggots. The figure represents relative expression values (REV) of the transcripts [(**A**) APAF1-interacting protein (**B**) Serine-threonine phosphatase (**C**) Cytochrome oxidase I (**D**) Caspase (**E**) C-type lectin (**F**) Tetraspanin 139 (**G**) Inositol polyphosphate multikinase (**H**) Cyclophilin (**I**) MAPK interacting serine-threonine kinase (**J**) Adenylate cyclase.] in maggots feeding on susceptible (SH; light bars) and resistant host (RH; shaded bars). Error bars represent Mean ± S.D.

Of the 10 genes selected for qPCR, five genes (APAF1-interacting protein, caspase, c-type lectin, cytochrome oxidase 1, and serine-threonine phosphatase) (Figure 6A–E) were up-regulated in maggots feeding on RH in comparison to maggots feeding on SH. It is to be noted that APAF1-interacting protein, caspase and cytochrome oxidase 1 have been reported to be constituents of the apoptosome complex in the apoptotic pathway. Up-regulation of genes involved in apoptotic pathway in maggots feeding on RH relate to the increased oxidative challenge encountered by the maggots in the resistant hosts. Expression of antioxidant genes in maggots feeding on SH was also observed though not to the same levels as those feeding on RH. This observation suggests that while feeding on SH the maggots have to encounter host oxidative defense mechanism, but probably not of the same severity while feeding on RH. These results are in accordance with results obtained from earlier studies on the Hessian fly-wheat interaction [43]. Therefore, future investigations should aim to unveil the mechanism in the maggots involved in detoxification of reactive oxygen compounds produced by the host. The remaining five genes (adenylate cyclase, cyclophilin, inositol polyphosphate multikinase, MAPK interacting serine-threonine kinase and tetraspanin 139) were down-regulated (Figure 6F–J) in maggots feeding on RH. Adenylate cyclase, cyclophilin and tetraspanin have been reported as conserved fungal virulence genes [37]. Down-regulation of genes reported as virulence factors in maggots feeding on RH is an interesting observation that should be further investigated to determine the functional role of these genes in gall midge-rice interaction.

## 3. Experimental Section

### 3.1. Collection of *Orseolia oryzae* Larvae and RNA Isolation

Gall midge biotype 4 (GMB4) used in this study is being cultured at the Directorate of Rice Research, Hyderabad, India under standard conditions [47]. The maggots were isolated from the rice varieties TN1 and Suraksha. GMB4 is virulent on TN1 (susceptible to GMB4; compatible interaction; SH) and avirulent on Suraksha (resistant to GMB4; incompatible interaction; RH). The maggot completes its lifecycle in the susceptible rice variety (TN1) whereas in the resistant variety (Suraksha) the maggot dies within 96 h post hatching. The first instar maggots are the feeding stages of the gall midge and resistance and susceptibility of the host is determined within this time frame. The first instar maggots were collected individually from the susceptible rice cultivar TN1 and Suraksha using entomological needles and stored in RNAlater (Qiagen, USA) for further use. Care was taken not to injure the maggots. Approximately 25,000 maggots from each rice variety (TN1 and Suraksha) were collected for the whole experiment.

### 3.2. RNA Isolation and 454 Sequencing

Maggots feeding on susceptible and resistant rice varieties were collected separately and total RNA was isolated using RNAeasy kit (Qiagen, USA) according to the manufacturer’s instructions. Quality and quantity of the RNA was assessed using Bioanalyzer (Agilent, Santa Clara, CA, USA). Two hundred micrograms of total RNA was used to isolate mRNA from both the samples using Oligotex mRNA isolation kit (Qiagen GmbH, Germany). Libraries were constructed and sequenced using Roche 454 GS-FLX system for both the samples separately, using the approach described earlier [15]. The library preparation and sequencing was performed by MWG Biotech, Germany. All the raw sequences generated have been deposited at the Sequence Read Archive at National Centre for Biotechnology Information with NCBI accession numbers SRA053211.

### 3.3. Transcript Assembly and Data Processing

All the raw sequences were adaptor-trimmed and sequences shorter than 50 bp were excluded from further analysis. Low complexity and mono/poly nucleotides containing reads were removed from the sequences. High quality reads that were considered for analysis contained more than 70% of high quality bases. Reads from both the samples were shuffled and merged together to generate a comprehensive transcripts assembly using MIRA 3 software [48]. The total number of sequences, mean length of all sequences, and number of contigs, average contig length, number of singlets were calculated. Separate assembly was also generated for both the samples. After assembly all these contigs and singlets were searched against the UniRef90 database (release 2011_8) using BLASTX [17] with a cut off value of *E* = 10^−3^. The same procedure was followed for both the assemblies. BLASTX was also performed with the NCBI-nr database and the samples were filtered with the same cut off as mentioned above. After performing BLASTX analysis and annotation of the sequences, sequences specific for library of GMB4 from SH and GMB4 from RH were separated. Gene ontology terms were assigned to contigs from both libraries and singlets identified using BLAST2GO tool [49] for all the annotated transcripts, annotated transcripts specifically present in GMB4 from SH, annotated transcripts specifically present in GMB4 from RH and annotated transcripts commonly present in both maggots from SH and RH. Metabolic pathway analysis using KEGG server [18] was performed for transcripts from SH and RH maggots. BLASTN analysis was also performed with the sequences from the Hessian fly (*Mayetiola destructor*) (available at NCBI database).

Microsatellite identification was performed using MISA [50] and the primers specific to the microsatellites were designed using Primer 3 [51]. The assembled contigs were analysed for SNPs as well (The reference sequence was assigned based on a contig formed by the largest numbers of individual sequences. Therefore, the reference sequence mentioned here is based on frequency rather than on a reference genome). The above in silico analyses were performed with assistance from M/s Bionivid Technology [P] Ltd., Bangalore, India.

### 3.4. Real-time PCR and Statistical Analyses

Quantitative Real Time PCR (qPCR) was carried out using total RNA isolated from the maggots feeding on SH and RH. Equal quantities of total RNA (as estimated by NanoVue, GE Healthcare, USA) was used for first strand cDNA synthesis using Superscript III Reverse Trancriptase (Invitrogen, USA) and oligodT primers according to the manufacturer’s protocol. qPCR primers were designed using Primer Express (Applied Biosystems, CA, USA) software (Table 6). Quantitative PCR was performed on Applied Biosystem StepOne Real-Time PCR system. The 20-μL of PCR mix contained cDNA (22 ng), 1× Power SYBR Green PCR mix (Applied Biosystems) and 0.5 mM of the primers. Cycling conditions were: 95 °C for 10 min followed by 40 cycles of 95 °C for 15 s and 60 °C for 1 min. Expression level was displayed as relative expression value based on the relative standard curve method. Results were analysed using 2^−ΔΔCt^ method built into the StepOnePlus Real-Time PCR analysis software (Applied Biosystems, USA) provided with the instrument. The relative expression values in the maggots feeding on SH were used as calibrator. Actin (GenBank accession number: JG450221) was included as the internal control for all the real-time PCR assays reported here. Actin was selected as the internal control after screening a set of other housekeeping genes using GeNorm software [52] and as reported in our earlier study [9]. Melt curve analysis was also performed. Statistical significance of the difference in mean was performed using Student’s *t*-test analysis [53]. Two biological and three technical replicates were included for the entire study.

## 4. Conclusions

Understanding the molecular basis of plant-insect interaction is useful not only for the biologists with evolutionary perspectives but also for researchers involved in pest management. This whole transcriptome study employing next generation sequencing technology revealed various metabolic pathways that are crucial in insect survival in RH and in modulating plant physiology in SH. Future studies directed towards functional validation of differentially expressed genes of target pathways would decipher the mechanism of insect virulence. Also, the molecular markers generated from the study will enable development of diagnostic tools for biotype differentiation and population variability analysis. Furthermore, these studies will provide major molecular resource for both plant biologists and entomologists to understand plant-insect interaction which in turn will help to develop better-integrated pest management strategies.

## Supplementary Materials



## Figures and Tables

**Table 1:** General features of expressed sequences tags (ESTs) libraries from virulent and avirulent Asian rice gall midge (GMB4) maggots.

	Virulent GMB4	Avirulent GMB4
Total number of sequence (bp) after filtering	79,028 (76,963) *	85,395 (84,118) *
High quality reads (%) **	70.25	72.44
Average reads length (bp)	242	236
Sequence length (%)	-	-
<100	3.01	1.67
101–500	96.63	98.03
501–1000	0.35	0.30
1001–1500	0.01	0
Number of contigs	9,272	9,526
Number of singletons	21,764	24,625

* Figures in parenthesis are reads with ≥70% high quality bases (phred score ≥ 20);

** Filtering sequences with low complexity and reads having length <50.

**Table 2:** List of transcripts identified in the Asian rice gall midge showing similarity to known proteins from other insects.

Functions	Code of ESTs	E-value	Similarity
**Melanization**			
	ICGEB_c3627	4 × 10^−48^	Dihydropteridine reductase
	ICGEB_rep_c3461	50	Prophenoloxidase
	FKB78SY06DOCUN	7 × 10^−18^	Serpin
	FKB78SY16JUF7R	3 × 10^−5^	Serpin 1
	FKB78SY14ITCIU	1.10 × 10^−3^	Laccase-4
**Immune regulatory proteins**			
	FKB78SY09FO3VQ	2 × 10^−10^	Toll
	FKB78SY15I6036	2 × 10^−9^	Intermediate in Toll signalling pathway
	ICGEB_rep_c1724	9 × 10^−70^	Ubiquitin carrier protein
	FKB78SY10GAGBA	1 × 10^−14^	GH14989
	ICGEB_rep_c1629	1 × 10^−47^	Histone H2A
	ICGEB_rep_c599	2 × 10^−8^	Transcription factor NFAt subunit NF45
	ICGEB_rep_c25	6 × 10^−22^	C-type lectin, galactose-binding
	ICGEB_c3769	1 × 10^−5^	Galactose-specific C-type lectin
	ICGEB_c2381	2 × 10^−32^	C-type lectin
	ICGEB_c10863	4 × 10^−2^	Lysozyme c-1
	FKB78SY15JHPUN	6.2 × 10^−3^	Argonaute 2
**Proteases**			
	ICGEB_rep_c638	6 × 10^−25^	Cathepsin L precursor
	ICGEB_rep_c1661	6 × 10^−9^	Putative gut cathepsin d-like aspartic protease
	ICGEB_c2413	8 × 10^−9^	Cathepsin l-like cysteine proteinase
	ICGEB_rep_c250	3 × 10^−6^	Cathepsin l-like cysteine proteinase CAL1
	ICGEB_rep_c1725	1 × 10^−39^	Chymotrypsin-like serine protease
	ICGEB_rep_c3018	4 × 10^−39^	Serine protease P100
	ICGEB_c3187	1 × 10^−35^	Serine protease H2
	ICGEB_c5978	1 × 10^−22^	CLIP-domain serine protease subfamily D
	ICGEB_c2987	7 × 10^−13^	Aspartic protease
	ICGEB_rep_c1876	3 × 10^−11^	Digestive cysteine protease
	ICGEB_rep_c9287	6 × 10^−11^	Signal peptide protease
	ICGEB_c4650	3 × 10^−9^	Serine protease htra2
	ICGEB_rep_c1661	6 × 10^−9^	Putative gut cathepsin d-like aspartic protease
	ICGEB_c6152	2 × 10^−8^	Lysosomal aspartic protease
	FKB78SY06DMP3X	4 × 10^−3^	Serine protease snake, putative
**Kinase**			
	FKB78SY14IS5EN	9 × 10^−34^	Serine-threonine kinase receptor-associated protein
	FKB78SY14IKKDH	5 × 10^−30^	Dual specificity MAPKK4
	ICGEB_c2935	2 × 10^−26^	MAPK kinase 1-interacting protein 1
	ICGEB_c8827	2 × 10^−22^	Inositol polyphosphate multikinase
	ICGEB_c8292	6 × 10^−22^	Casein kinase II subunit alpha
	FKB78SY09FSJ0Q	9 × 10^−16^	Serine-threonine kinase
	FKB78SY11GZ55Y	3 × 10^−18^	Src tyrosine kinase, putative
	ICGEB_c8214	6 × 10^−20^	Nucleoside diphosphate kinase
**Apoptosis and cell death**			
	ICGEB_c2475	7 × 10^−13^	Caspase long class, Dronc-like
	FKB78SY07D8PWI	4 × 10^−14^	Inhibitor of apoptosis 2 protein
	ICGEB_c3186	1 × 10^−28^	Defender against apoptotic cell death
	ICGEB_rep_c1321	3 × 10^−6^	Programmed cell death protein 7
	ICGEB_c10998	6 × 10^−4^	Programmed cell death 4a
**ROS related genes**			
	ICGEB_c8627	7 × 10^−12^	Catalase
	ICGEB_rep_c11026	1 × 10^−21^	Superoxide dismutase [Cu-Zn]
	ICGEB_rep_c991	6 × 10^−40^	Glutathione *S*-transferase
	ICGEB_c5428	4 × 10^−38^	Glutathione peroxidase
	ICGEB_c4435	6 × 10^−11^	Cytochrome P450

**Table 3:** Putative microsatellite loci predicted in the Asian rice gall midge.

Microsatellite Repeats	Number of loci
Dincucleotide	2,380
Trinucleotide	1,205
Tetranucleotide	134
Pentanucleotide	56
Hexanucleotide	300
Complex	143
Total	4,218

**Table 4:** Types of putative single nucleotide polymorphism (SNPs) identified in *Orseolia oryzae* transcriptome.

SNP type	Counts
**Transition**	
A-G	282
C-T	120
**Transversion**	
A-C	300
A-T	390
C-G	49
G-T	101
Total	1242
**Others** *	1514
Grand Total	2756

* Includes K/(G/T), M/(A/C), R/(A/G), S/(G/C), W/(A/T), Y/(C/T), A/(C/G/T), T/(A/C/G), C/(A/T/G), G/(A/T/C).

**Table 5:** Top 15 contigs commonly and uniquely present in virulent and avirulent GMB4.

Specifically present in virulent GMB4					Specifically present in avirulent GMB4				Commonly present in both interaction			

Contig id	Similarity	CL	*N*	Contig id	Similarity	CL	N	Contig id	Similarity	CL	*N*	
											
											Virulent	Avirulent
ICGEB_rep_c1075	Zinc finger protein	128	18	ICGEB_c2372	Serine-threonine protein phosphatase	176	11	ICGEB_rep_c6546	Tropomyosin-2	164	366	449
ICGEB_rep_c1403	AGAP008060-PA	302	10	ICGEB_c3178	Piopio protein	281	8	ICGEB_rep_c43	Predicted protein	464	133	484
ICGEB_rep_c1433	Putative uncharacterized protein	187	9	ICGEB_c2916	Putative uncharacterized protein	326	8	ICGEB_rep_c128	Ribosomal protein L37	251	470	375
ICGEB_rep_c1962	GF23525	80	8	ICGEB_rep_c1359	Pseudouridine synthase	113	8	ICGEB_rep_c21	60S ribosomal protein L24	232	319	277
ICGEB_c2217	Proteasome subunit beta type	100	8	ICGEB_c2037	Putative uncharacterized protein	287	7	ICGEB_rep_c12	ATP synthase subunit a	429	211	358
ICGEB_c2817	Putative uncharacterized protein	182	7	ICGEB_rep_c4187	Putative uncharacterized protein	107	7	ICGEB_rep_c2	Midline fasciclin	167	306	229
ICGEB_c2608	Ribosomal protein S25	96	7	ICGEB_rep_c3796	Ribosomal protein, L48, putative	200	7	ICGEB_rep_c24	GH15515	257	240	186
ICGEB_c2406	FKBP-type peptidylprolyl cis-trans isomerase	164	7	ICGEB_c2559	GF20993	173	6	ICGEB_rep_c19	60S ribosomal protein L23	290	203	201
ICGEB_c2935	Mitogen-activated protein kinase kinase 1-interacting protein 1	284	7	ICGEB_c2421	GL24166	185	6	ICGEB_rep_c10	Cytochrome C oxidase subunit 1	229	238	194
ICGEB_c2896	Multiple coagulation factor deficiency protein 2-like protein	222	7	ICGEB_c2757	AGAP004322-PA (Fragment)	164	6	ICGEB_rep_c82	Cytochrome b	224	217	223
ICGEB_rep_c2089	APAF1-interacting protein-like protein	170	7	ICGEB_rep_c4363	GI18785	208	5	ICGEB_rep_c10597	60S ribosomal protein L12	217	245	97
ICGEB_rep_c2623	Putative uncharacterized protein	135	7	ICGEB_c4321	GJ10149	80	5	ICGEB_rep_c54	Ribosomal protein L19	326	212	140
ICGEB_c2915	Putative uncharacterized protein	100	7	ICGEB_c10901	Putative uncharacterized protein	95	5	ICGEB_rep_c3468	NAD-Hubiquinone oxidoreductase chain 4	347	193	155
ICGEB_c2381	C-type lectin	212	7	ICGEB_c2603	Putative uncharacterized protein	110	5	ICGEB_rep_c25	AGAP010193-PA	1139	174	156
ICGEB_c2477	GA11576	206	7	ICGEB_rep_c2725	GA19828	269	5	ICGEB_rep_c107	GL12416	137	214	110

CL: Contig length; N: Frequency of transcripts.

**Table 6:** List of genes selected and sequence of the primers used for RT-PCR profiling.

S. No	Gene name	Primer name	Primer sequence (5′-3′)	*T*_m_ (°C)	Amplicon size (bp)
1	*Cytochrome oxidase I*	RTCytoc F	TGTAGGAATAGAAGTTGATACACGAGCTT	60	185
		RTCytoc R	CTCCTGTCACTCCTCCAATAGTAAATAA		

2	*Serine-threonine phosphatase*	RTSTP F	TAAAGACATGCGAGGGTGAGAGT	60	120
		RTSTP R	CAGCGACAGAAAATGGTGACA		

3	*APAF1-interacting protein*	RTAIP1 F	GTTCGCCGACACGGACTTTA	60	104
		RTAIP1 R	TTCTTCATTTCCACAGCAATTCC		

4	*Cyclophilin*	RTCyclop F	GGTATTTTTGGATATGTCGTCGAA	60	102
		RTCyclop R	GCTGCTGATTATCACATTACTTTGTG		

5	*Tetraspanin 139*	RTTet139 F	TCACCATCCGAATGGATTCC	60	129
		RTTet139 R	CCCGCTGCCAATCAATTCT		

6	*Adenylate cyclase*	RTAdcycl F	GAGGCCCGGCAAAGAAGA	60	100
		RTAdcycl R	AGCGAGTGCAAATTCCACAAC		

7	*MAPK interacting serine-threonine kinase*	RTMAPKistk F	CTGAAAGCGAAAATGCCGATA	60	100
		RTMAPKistk R	CTCAATTCACGTGCCGATTG		

8	*C-type lectin*	RTCTL F	CGGTGCCCACGAAAACTG	60	131
		RTCTL R	GCACATATTTCAGAAGTGCATCATT		

9	*Inositol polyphosphate multikinase*	RTIPMK F	GAGAATGGGCCTATGTCAAAATG	60	101
		RTIPMK R	CACAAGATTTTCGATGCCAAATAA		

10	*Caspase*	RTCasp F	AAACGAGTAGTGAAGGTGCAAACATA	60	150
		RTCasp R	CGTGCGCATGTTCAGCTAAT		
