# Supplementary material for: Pyrosequencing-Based Transcriptome Analysis of the Asian Rice Gall Midge Reveals Differential Response during Compatible and Incompatible Interaction
Source: Int J Mol Sci. 2012 Oct 12;13(10):13079–103. doi: 10.3390/ijms131013079 (PMC3497313; doi:10.3390/ijms131013079)
Supplement: Supplementary file 1 [file ijms-13-13079-s001.pdf]

Supplementary Information

Figure S1 (**A**) Visualization of the transcripts involved in 'aminoacyl-tRNA biosynthesis' pathway in the rice gall midge maggots isolated from SH. Genes identified in the present study are highlighted; (**B**) Visualization of the transcripts involved in 'aminoacyl-tRNA biosynthesis' pathway in the rice gall midge maggots isolated from RH. Genes identified in the present study are highlighted.

A

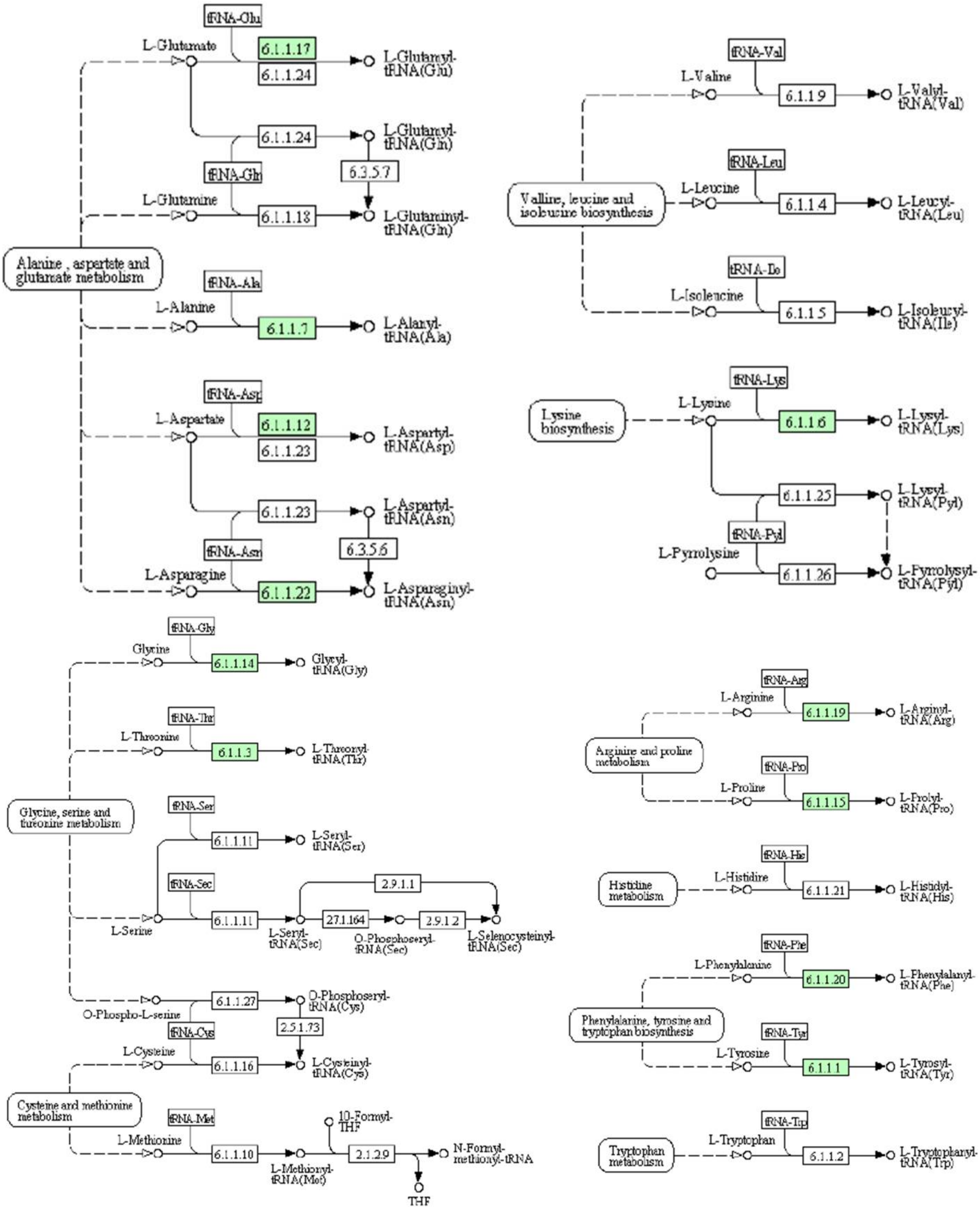

Figure S1. *Cont.*

B

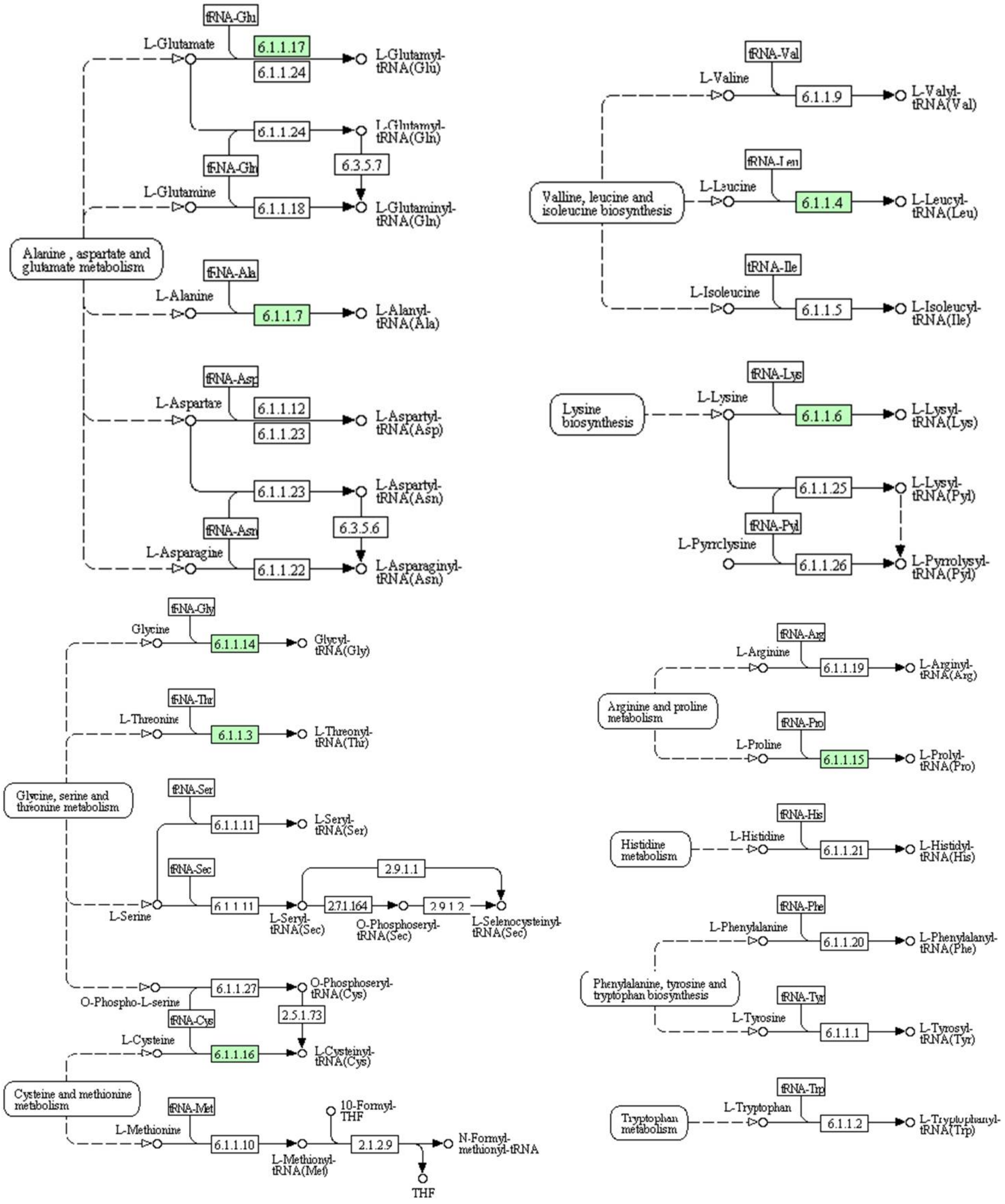

Figure S2. (**A**) Visualization of the transcripts involved in 'oxidative phosphorylation' pathway in the rice gall midge maggots isolated from SH. Genes identified in the present study are highlighted; (**B**) Visualization of the transcripts involved in 'oxidative phosphorylation' pathway in the rice gall midge maggots isolated from RH. Genes identified in the present study are highlighted.

A

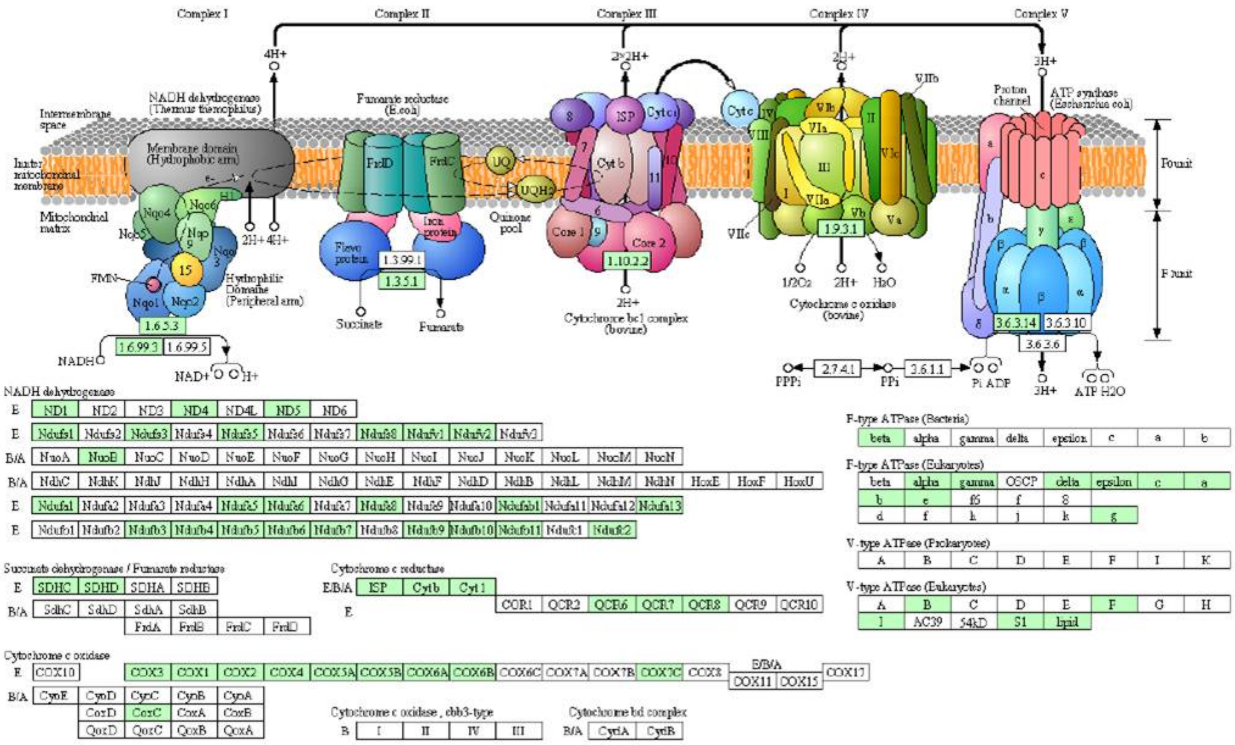

B

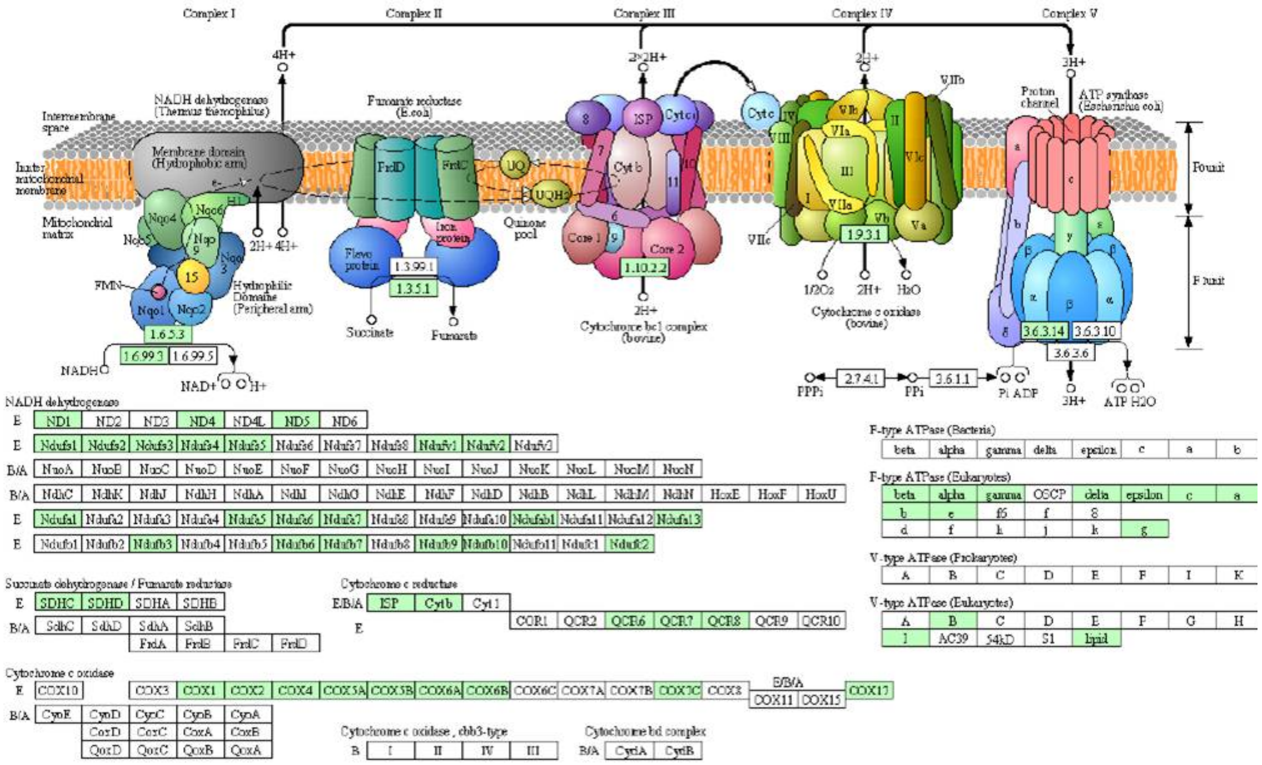

Figure S3. Venn diagram representing common and specifically present genes in the Asian rice gall midge maggots isolated from SH and RH. Numbers represent genes identified to be specifically or commonly present in the maggots from SH and RH.

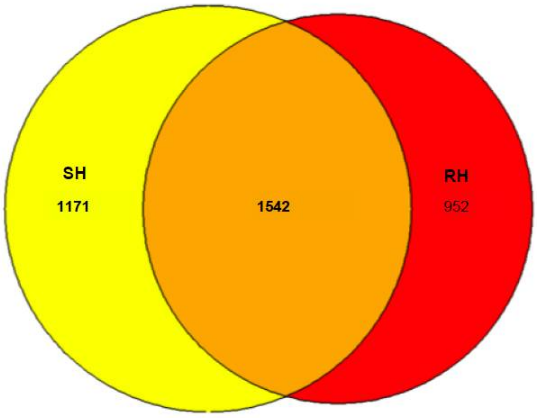

© 2012 by the authors; licensee MDPI, Basel, Switzerland. This article is an open access article distributed under the terms and conditions of the Creative Commons Attribution license (http://creativecommons.org/licenses/by/3.0/).
